# Supplementary material for: Glufosinate constrains synchronous and metachronous metastasis by promoting anti‐tumor macrophages
Source: EMBO Mol Med. 2020 Sep 4;12(10):e11210. doi: 10.15252/emmm.201911210 (PMC7539200; doi:10.15252/emmm.201911210)
Supplement: Supplementary file 1 — Appendix [file EMMM-12-e11210-s001.pdf]

Glufosinate constrains synchronous and metachronous metastasis by promoting anti-tumor macrophages

Appendix

Contents

Appendix Table S1. List of reagents

Appendix Table S2. P values and statistical test for all figures

Appendix Table S1- List of reagents

Reagent	Source	Identifier	Diluition
anti-mouse CD16/CD32	BD Biosciences	553142	1/200
anti-CD4	BioLegend	100540	1/200
anti-CD8	eBioscience	53-0081-82	1/200
anti-MHCII	eBioscience	46-5321-82	1/200
anti-CD206	BioLegend	321120	1/100
anti-CD11c	eBioscience	17-0114-81	1/100
anti-CD69	BioLegend	104522	1/50
anti-CD45	BioLegend	103108	1/50
anti-F4/80	BioLegend	123128	1/100
anti-TCRbeta	Thermo Fisher Scientific	17-5796-82	1/100
anti-CD25	eBioscience	25-0251-82	1/100
rat anti-CD31	BD Biosciences	557355	1/200
rat anti-F4/80	Bio-Rad	MCA497G	1/100
rabbit anti-hypoxyprobe	NPS	HP3-100KIT	1/100
rat anti-Thy1.1	Abcam	ab85352	1/200
rat anti-actin α-smooth muscle	Sigma-Aldrich	C6198-.2ML	1/500
goat anti-CD206	R&D Systems	AF2535	1/100
rabbit anti-CA9	Novus Biologicals	NB100-417	1/100
rabbit anti-FITC	Biorad	4510-7604	1/100
hamster anti-CD11c	eBioscience	14-0114-81	1/200
Alexa FluoA22:A40r® 488 anti-rat CD90/mouse CD90.1 (Thy-1.1) [OX-7] 100 µg	BioLegend	202506	1/200
Donkey anti-Rabbit IgG (H+L) Secondary Antibody, Alexa Fluor® 488 conjugate	Thermo Fisher Scientific	# A-21206	1/500
anti-GFP (488)	Invitrogen	A21311	1/200
Donkey anti-Rat IgG (H+L) Secondary Antibody, Alexa Fluor® 488 conjugate	Life Technologies	A21208	1/200
Donkey anti-Goat IgG (H+L) Secondary Antibody, Alexa Fluor® 488 conjugate	Invitrogen	A-11055	1/200
Donkey anti-Mouse IgG (H+L) Secondary Antibody, Alexa Fluor 488	Thermo Fisher	A32766	1/200
Goat anti-Rabbit IgG (H+L) Cross-Adsorbed Secondary Antibody, Alexa Fluor 488	Thermo Fisher	A11008	1/200
Streptavidin, Alexa Fluor® 488 conjugate	Thermo Fisher	S11223	1/200
Goat anti-Rat IgG (H+L) Secondary Antibody, Alexa Fluor 488	Thermo Fisher	A-11006	1/200
Donkey anti-Goat IgG (H+L) Secondary Antibody, Alexa Fluor® 568 conjugate	Life Technologies	A-11057	1/200
Donkey anti-Mouse IgG Secondary Antibody, Alexa Fluor® 568 conjugate	Thermo Fisher	A10037	1/200
goat anti rat alexa 568	LifeTechnology	A11077	1/200
Goat anti-Rabbit IgG (H+L) Secondary Antibody, Alexa Fluor 568	Thermo Fisher	A11036	1/200
Donkey anti-Rabbit IgG (H+L) Secondary Antibody, Alexa Fluor 568	Thermo Fisher	A10042	1/200
ANTI-ACTIN ALPHA-SMOOTH MUSCLE - CY3&	Sigma-Aldrich	C6198-.2ML	1/100
biotin-labeled antibody	bio-connect	711-065-152	1/300
biotin-labeled antibody	bio-connect	705-065-003	1/300
Goat anti-Rat IgG (H+L) Cross-Adsorbed Secondary Antibody, Alexa Fluor 555	Thermo Fisher	A-21434	1/200
Goat anti-Rabbit IgG (H+L) Cross-Adsorbed Secondary Antibody, Alexa Fluor 405	Thermo Fisher	A-31556	1/200
TSA Plus Fluorescein	Perkin Elmer, Life Sciences	NEL741B001KT	
TSA Plus Cyanine 3	Perkin Elmer, Life Sciences	NEL744B001KT	
FIH-1/HIF-1AN	Novus Biologicals	NB100-428	1/500
Phospho-4E-BP1 (Thr37/46)	Cell Signaling	#2855	1/500
4E-BP1	Cell Signaling	#4923	1/500
p70 S6 Kinase Antibody	Cell Signaling	#9202	1/1000
Phospho-p70 S6 Kinase (Thr389)	Cell Signaling	#9206	1/1000
REDD-1 (N-20)	Santa Cruz Biotechnology	sc-46034	1/500
S6 Ribosomal Protein (5G10) Rabbit	Cell Signaling	#2217	1/1000
Phospho-S6 Ribosomal Protein (Ser235/236)	Cell Signaling	#4858	1/1000
Anti-TNF alpha	Abcam	ab9635	1/1000
Anti-CCL18 antibody	Novus	NBP1-79940	1/1000
Anti-Glutamine Synthetase	Sigma-Aldrich	MAB302	1/1000
Monoclonal Anti-Vinculin antibody produced in mouse	sigma	V9131-.2ML	1/1000
anti-CSF-1R antibody	BioXcell	clone AFS98	
Lectin FITC labeled	VectorLab	FL-1171	
ProLong® Gold Antifade Mountant with DAPI	Life Technologies	P36935	
Hoechst 33342	Thermo Fisher Scientific	H3570	
Calcein AM	Thermo Fisher Scientific	C3099	
Rat IgG2a Negative Control	sigma	MABF1077Z	
Mouse Interferon gamma ELISA Kit	Abcam	ab46081	
GLUFOSINATE-AMMONIUM PESTANAL (AMMONIUM-	sigma	45520-100MG	
DL-PHOSPHINOTRICIN	Santa Cruz Biotechnology	sc-263102	
real-time PCR probes:			
Ccl22	IDT	Mm.PT.58.15758573	
Murine Arg1 primer exons 3-4	IDT	Mm.PT.58.8651372	
NOS2	IDT	Mm.PT.58.43705194	
Ccl17	IDT	Mm.PT.58.9199490	
TNFa	IDT	Mm.PT.56a.29509614	
CxcL9	IDT	Mm.PT.58.5726745	
CxcL10	IDT	Mm.PT.58.43575827	
CD86	IDT	Mm.PT.58.45949515	
CD80	IDT	Mm.PT.58.10255942	
GLUL	Thermo Fisher Scientific	Hs00365928_g1	
CD80	Thermo Fisher Scientific	Hs01045161_m1	
CXCL9	Thermo Fisher Scientific	Hs00171065_m1	
CXCL10	Thermo Fisher Scientific	Hs00171042_m1	
TNFa	Thermo Fisher Scientific	Hs00174128_m1	
MRC1	Thermo Fisher Scientific	Hs00267207_m1	
MSR1	Thermo Fisher Scientific	Hs00234007_m1	
CCL17	Thermo Fisher Scientific	Hs00171074_m1	
CCL18	Thermo Fisher Scientific	Hs00268113_m1	
CD86	Thermo Fisher Scientific	Hs01567026_m1	

Table S2. P values and statistical test for all figures

Figure 2	2A	P value	Statistical test
	IL10 vs IL10/MSO	0,032000	one way ANOVA
	IL10 vs IL10/Gluf 10	0,009240	one way ANOVA
	IL10 vs IL10/Gluf 20	0,000900	one way ANOVA
	2B	Pvalue	statistical test
	IL10 vs IL10/MSO		one way ANOVA
	IL10 vs IL10/Gluf 10	0,003400	one way ANOVA
	IL10 vs IL10/Gluf 20	0,000430	one way ANOVA
	2C	P value	Statistical test
	IL10 vs IL10/MSO	0,009700	one way ANOVA
	IL10 vs IL10/Gluf 10	0,005400	one way ANOVA
	IL10 vs IL10/Gluf 20	0,000240	one way ANOVA
	2D	P value	Statistical test
	IL10 vs IL10/MSO	0,023400	one way ANOVA
	IL10 vs IL10/Gluf 10		one way ANOVA
	IL10 vs IL10/Gluf 20	0,000750	one way ANOVA
	2E	P value	Statistical test
	IL10 vs IL10/MSO	0,043200	one way ANOVA
	IL10 vs IL10/Gluf 10	0,004800	one way ANOVA
	IL10 vs IL10/Gluf 20	0,022200	one way ANOVA
	2F	P value	Statistical test
	IL10 vs IL10/MSO	0,007500	one way ANOVA
	IL10 vs IL10/Gluf 10	0,003210	one way ANOVA
	IL10 vs IL10/Gluf 20	0,000450	one way ANOVA
	2G	P value	Statistical test
	IL10 vs IL10/MSO	0,012300	one way ANOVA
	IL10 vs IL10/Gluf 10	0,043200	one way ANOVA
	IL10 vs IL10/Gluf 20	0,023200	one way ANOVA
	2H	P value	Statistical test
	IL10 vs IL10/MSO	0,044890	one way ANOVA
	IL10 vs IL10/Gluf 10	0,027860	one way ANOVA
	IL10 vs IL10/Gluf 20	0,033670	one way ANOVA
	2I	P value	Statistical test
	IL10 vs IL/Gluf10	0,005640	one way ANOVA
	IL10 vs IL10/Gluf 20	<0,0001	one way ANOVA
	IL10 Gluf10 vs IL10 Gluf10/acr	0,015674	one way ANOVA
	IL10 Gluf20 vs IL10 Gluf20/acr	<0,0001	one way ANOVA
	2J	P value	Statistical test
	IL10 vs IL/Gluf10	0,036840	one way ANOVA
	IL10 vs IL10/Gluf 20	0,007456	one way ANOVA
	IL10 Gluf10 vs IL10 Gluf10/acr	0,000336	one way ANOVA
	IL10 Gluf20 vs IL10 Gluf20/acr	<0,0001	one way ANOVA
	2K	P value	Statistical test
	IL10 vs IL/Gluf10	0,000476	one way ANOVA
	IL10 vs IL10/Gluf 20		one way ANOVA
	IL10 Gluf10 vs IL10 Gluf10/acr	<0,0001	one way ANOVA
	IL10 Gluf20 vs IL10 Gluf20/acr	0,008750	one way ANOVA
	2L	P value	Statistical test
	IL10 vs IL/Gluf10	<0,0001	one way ANOVA
	IL10 vs IL10/Gluf 20	<0,0001	one way ANOVA
	IL10 Gluf10 vs IL10 Gluf10/acr	<0,0001	one way ANOVA
	IL10 Gluf20 vs IL10 Gluf20/acr	<0,0001	one way ANOVA
	2M	P value	Statistical test
	IL10 vs IL/Gluf10	0,000577	one way ANOVA
	IL10 vs IL10/Gluf 20	0,000340	one way ANOVA
	IL10 Gluf10 vs IL10 Gluf10/acr	0,000365	one way ANOVA
	IL10 Gluf20 vs IL10 Gluf20/acr	<0,0001	one way ANOVA
	2N	P value	Statistical test
	IL10 vs IL/Gluf10	0,003756	one way ANOVA
	IL10 vs IL10/Gluf 20		one way ANOVA
	IL10 Gluf10 vs IL10 Gluf10/acr	0,004435	one way ANOVA
	IL10 Gluf20 vs IL10 Gluf20/acr	0,006865	one way ANOVA
	2O	P value	Statistical test
	IL10 vs IL/Gluf10	0,000874	one way ANOVA
	IL10 vs IL10/Gluf 20	0,034560	one way ANOVA
	IL10 Gluf10 vs IL10 Gluf10/acr	<0,0001	one way ANOVA
	IL10 Gluf20 vs IL10 Gluf20/acr	0,012880	one way ANOVA
	2P	P value	Statistical test
	IL10 vs IL/Gluf10	0,000670	one way ANOVA
	IL10 vs IL10/Gluf 20	0,007464	one way ANOVA
	IL10 Gluf10 vs IL10 Gluf10/acr	0,000546	one way ANOVA
	IL10 Gluf20 vs IL10 Gluf20/acr	0,000356	one way ANOVA

	2Q	P value	Statistical test
	IL10 vs IL10/MSO	<0,0001	one way ANOVA
	IL10 vs IL10/Gluf 10	<0,0001	one way ANOVA
	IL10 vs IL10/Gluf 20	<0,0001	one way ANOVA
	IL10/MSO vs IL10/Gluf 20	0,006465	one way ANOVA
	IL10/Gluf 10 vs IL10/Gluf 20	0,003245	one way ANOVA
	2R	P value	Statistical test
	IL10 vs IL10/MSO	<0,0001	one way ANOVA
	IL10 vs IL10/Gluf 10	<0,0001	one way ANOVA
	IL10 vs IL10/Gluf 20	<0,0001	one way ANOVA
	IL10/MSO vs IL10/Gluf 20	0,006674	one way ANOVA
	IL10/MSO vs IL10/Gluf 10	0,034560	one way ANOVA
	IL10/Gluf 10 vs IL10/Gluf 20	0,024599	one way ANOVA
	2S	P value	Statistical test
	IL10 vs IL10/MSO	0,029980	one way ANOVA
	IL10 vs IL10/Gluf 10	0,028955	one way ANOVA
	IL10 vs IL10/Gluf 20	0,007774	one way ANOVA
	2T	P value	Statistical test
	Mo vs CXCL10	<0,0001	one way ANOVA
	Mo vs LPS/IFN	0,000864	one way ANOVA
	CXCL10 vs IL10	<0,0001	one way ANOVA
	LPS vs IL10	<0,0001	one way ANOVA
	IL10 vs IL10/MSO	0,032450	one way ANOVA
	IL10/MSO vs IL10/Gluf10	0,049980	one way ANOVA
	IL10 vs IL10/Gluf 10	<0,0001	one way ANOVA
	IL10 vs IL10/Gluf 20	<0,0001	one way ANOVA
	2V	P value	Statistical test
	IL10 (N) vs IL10/Gluf 20 (N)	0,000547	two way ANOVA
	IL10/Rap(N) vs IL10/Gluf 20 (N)	0,000854	two way ANOVA
	IL10/Gluf20(N) vs IL10/Gluf 20/Rap (N)	0,000344	two way ANOVA
	IL10 (H) vs IL10/Gluf 20 (N)	0,000635	two way ANOVA
	IL10 (H) vs IL10/Gluf 20 (H)	0,021223	two way ANOVA
	IL10/Rap(H) vs IL10/Gluf 20 (H)	0,043456	two way ANOVA
	IL10/Rap(H) vs IL10/Gluf 20 (N)	0,000877	two way ANOVA
	IL10/Gluf 20 (N) vs IL10/GLuf20 (H)	0,023334	two way ANOVA
	IL10/Gluf 20 (N) vs IL10/GLuf20/Rap(H)	0,000488	two way ANOVA
	IL10/Gluf20(H) vs IL10/Gluf 20/Rap (H)	0,049987	two way ANOVA
Figure 3	3B	P value	Statistical test
	10 vs 20	0,028550	one way ANOVA
	veh vs 20	0,007640	one way ANOVA
	veh vs 40	0,032400	one way ANOVA
	3C	P value	Statistical test
	veh vs 10	0,012346	one way ANOVA
	veh vs 20	0,032220	one way ANOVA
	3E	P value	Statistical test
	veh vs 10	0,027770	one way ANOVA
	veh vs 20	0,016764	one way ANOVA
	3F	P value	Statistical test
	veh vs 20	0,004360	two tailed *t* test
	3G	P value	Statistical test
	veh vs 20	0,000434	two tailed *t* test
	3H	P value	Statistical test
	veh vs 20	0,006453	two tailed *t* test
	3I-Ccl22	P value	Statistical test
	veh vs 10	0,006565	one way ANOVA
	veh vs 20	0,000764	one way ANOVA
	3I-Arg1	P value	Statistical test
	veh vs 10	0,045600	one way ANOVA
	veh vs 20	0,046880	one way ANOVA
	3I-Ccl17	P value	Statistical test
	veh vs 10	0,007640	one way ANOVA
	veh vs 20	0,023000	one way ANOVA
	3I-Tnfa	P value	Statistical test
	veh vs 10		one way ANOVA
	veh vs 20	0,011123	one way ANOVA
	3I-Cxcl9	P value	Statistical test
	veh vs 10		one way ANOVA
	veh vs 20	0,043350	one way ANOVA
	3I-Nos2	P value	Statistical test
	veh vs 10		one way ANOVA
	veh vs 20	0,035700	one way ANOVA
	3I-Cd80	P value	Statistical test
	veh vs 10		one way ANOVA
	veh vs 20	0,024000	one way ANOVA

	3I-Cd86	P value	Statistical test
	10 vs 20	0,043250	one way ANOVA
	veh vs 20	0,008655	one way ANOVA
Figure 4	4A	P value	Statistical test
	veh vs 20	<0,0001	two tailed *t* test
	4B	P value	Statistical test
	veh vs 20	<0,0001	two tailed *t* test
	4C	P value	Statistical test
	veh vs 20	0,033250	two tailed *t* test
	4D	P value	Statistical test
	veh vs 20	0,008655	two tailed *t* test
	4E	P value	Statistical test
	veh vs 20	<0,0001	two tailed *t* test
	4F	P value	Statistical test
	veh vs 20	0,043300	two tailed *t* test
	4G	P value	Statistical test
	veh vs 20	0,013555	two tailed *t* test
	4H	P value	Statistical test
	veh vs 20	0,006433	two tailed *t* test
	4I	P value	Statistical test
	veh vs 20	<0,0001	two tailed *t* test
Figure 5	5D	P value	Statistical test
	before t vs vehicle	0,043300	one way ANOVA
	veh vs 20	0,024500	one way ANOVA
	5D (<0.5mm2)	P value	Statistical test
	before t vs vehicle	0,000444	two way ANOVA
	veh vs 20	0,000644	two way ANOVA
	5D (1-1.5mm2)	P value	Statistical test
	before t vs vehicle	0,006400	two way ANOVA
	veh vs 20	0,000754	two way ANOVA
	5D (>1.5mm2)	P value	Statistical test
	before t vs vehicle	0,000743	two way ANOVA
	veh vs 20	0,000854	two way ANOVA
	5F	P value	Statistical test
	veh/IgG vs 20/IgG	0,043400	one way ANOVA
	20/IgG vs veh/AntiCSF1R	0,007640	one way ANOVA
	20/IgG vs 20/AntiCSF1R	<0,0001	one way ANOVA
Figure 6	6C	P value	Statistical test
	veh vs 20	0,008550	two tailed *t* test
	6E	P value	Statistical test
	veh vs 20	0,024400	two tailed *t* test
	6F	P value	Statistical test
	veh vs 20	0,008750	two tailed *t* test
	6G	P value	Statistical test
	veh vs 20	0,003677	two tailed *t* test
	6H	P value	Statistical test
	veh vs 20	0,022300	two tailed *t* test
	6I	P value	Statistical test
	veh vs 20	0,044600	two tailed *t* test
	6J	P value	Statistical test
	veh vs 20	0,006436	two tailed *t* test
	6K	P value	Statistical test
	veh vs 20	0,008870	two tailed *t* test
	6L	P value	Statistical test
	veh vs 20	0,003366	two tailed *t* test
	6M	P value	Statistical test
	veh vs 20	0,019990	two tailed *t* test
	6N Arg1	P value	Statistical test
	veh vs 20	0,012500	two tailed *t* test
	6N Cxcr4	P value	Statistical test
	veh vs 20	0,034420	two tailed *t* test
	6N Cxcl9	P value	Statistical test
	veh vs 20	0,049900	two tailed *t* test
	6N Nos2	P value	Statistical test
	veh vs 20	0,043200	two tailed *t* test
Figure 7	7A	P value	Statistical test
	veh vs 20	<0,0001	two tailed *t* test
	7B	P value	Statistical test
	veh vs 20	0,003330	two tailed *t* test
	7C	P value	Statistical test
	veh vs 20	0,039990	two tailed *t* test
	7D	P value	Statistical test
	veh vs 20	0,006330	two tailed *t* test
	7E	P value	Statistical test

	veh vs 20	0,029898	two tailed *t* test
	7F	P value	Statistical test
	veh vs 20	0,002255	two tailed *t* test
Figure 8	8D	P value	Statistical test
	veh vs 10	0,044560	two tailed *t* test
	8E	P value	Statistical test
	veh vs 10	0,000654	two tailed *t* test
	8F (<0.5mm2)	P value	Statistical test
	veh vs 10	<0,0001	two way ANOVA
	8F (>1.0mm2)	P value	Statistical test
	veh vs 10	<0,0001	two way ANOVA
	8I	P value	Statistical test
	veh vs 10	0,034460	two tailed *t* test
	8K	P value	Statistical test
	veh vs 10	0,043340	two tailed *t* test
	8L	P value	Statistical test
	veh vs 10	0,022400	two tailed *t* test
	8M	P value	Statistical test
	veh vs 10	0,012400	log rank (Mantel–Cox) test
	8O	P value	Statistical test
	veh vs 10	0,046540	two tailed *t* test
Figure 9	9C	P value	Statistical test
	veh vs 10	0,029990	one way ANOVA
	veh vs 20	0,019870	one way ANOVA
	9G	P value	Statistical test
	baseline vs day15	0,009970	two way ANOVA
Figure EV1	EV1A-Glutamate	P value	Statistical test
	IL10 vs IL10/Gluf 10	0,000637	one way ANOVA
	IL10 vs IL10/Gluf 20	0,000334	one way ANOVA
	IL10 Gluf10 vs IL10 Gluf20	0,037760	one way ANOVA
	EV1A-Glutamine	P value	Statistical test
	IL10 vs IL10/Gluf 10	0,004330	one way ANOVA
	IL10 vs IL10/Gluf 20	0,000764	one way ANOVA
	IL10 Gluf10 vs IL10 Gluf20	0,046660	one way ANOVA
	EV1A-Succinate	P value	Statistical test
	IL10 vs IL10/Gluf 10	0,006530	one way ANOVA
	IL10 vs IL10/Gluf 20	0,000320	one way ANOVA
	IL10 Gluf10 vs IL10 Gluf20	0,005430	one way ANOVA
Figure EV2	EV2A-Cxcl10	P value	Statistical test
	IL10 vs IL10/Gluf 10	0,000653	one way ANOVA
	IL10 vs IL10/Gluf 20	0,000655	one way ANOVA
	EV2A-Tnfa	P value	Statistical test
	IL10 vs IL10/Gluf 10	0,000112	one way ANOVA
	IL10 vs IL10/Gluf 20	0,000322	one way ANOVA
	EV2A-Nos2	P value	Statistical test
	IL10 vs IL10/Gluf 10	0,000643	one way ANOVA
	IL10 vs IL10/Gluf 20	0,000325	one way ANOVA
	EV2B-Arg1	P value	Statistical test
	IL10 Gluf10 vs IL10 Gluf20	0,004300	one way ANOVA
	EV2B-Ccl22	P value	Statistical test
	IL10 vs IL10/Gluf 10	0,000112	one way ANOVA
	IL10 vs IL10/Gluf 20	0,000329	one way ANOVA
	EV2C	P value	Statistical test
	Mo vs Mo/IL10	0,000543	one way ANOVA
	M0/IL10 vs M0/IL10/Gluf 10	<0,0001	one way ANOVA
	M0/IL10 vs M0/IL10/Gluf 20	<0,0001	one way ANOVA
	EV2D	P value	Statistical test
	M0/IL10 vs M0/IL10/Gluf 10	<0,0001	one way ANOVA
	M0/IL10 vs M0/IL10/Gluf 20	<0,0001	one way ANOVA
	EV2E-LLC proliferation	P value	Statistical test
	48h-Gln vs 48h+Gln	0,005430	one way ANOVA
	72h-Gln vs 72h+Gln	0,000543	one way ANOVA
	EV2E-LLC migration	P value	Statistical test
	veh +Gln vs veh -Gln	0,043300	one way ANOVA
	veh +Gln vs Gluf 10 -Gln	0,000654	one way ANOVA
	veh +Gln vs Gluf 20 -Gln	0,049990	one way ANOVA
	Gluf 10 +Gln vs veh -Gln	0,006540	one way ANOVA
	Gluf 10 +Gln vs Gluf 10 -Gln	0,000765	one way ANOVA
	Gluf 10 +Gln vs Gluf 20 -Gln	0,012990	one way ANOVA
	Gluf 20 +Gln vs veh -Gln	0,005330	one way ANOVA
	Gluf 20 +Gln vs Gluf 10 -Gln	0,000540	one way ANOVA
	Gluf 20 +Gln vs Gluf 20 -Gln	0,023500	one way ANOVA
	EV2F-migrated 4T1	P value	Statistical test
	veh +Gln vs veh -Gln	0,005500	one way ANOVA

	veh +Gln vs Gluf 10 -Gln	0,004320	one way ANOVA
	veh +Gln vs Gluf 20 -Gln	0,043300	one way ANOVA
	Gluf 20 +Gln vs veh -Gln	0,008650	one way ANOVA
	Gluf 20 +Gln vs Gluf 10 -Gln	0,000765	one way ANOVA
	Gluf 20 +Gln vs Gluf 20 -Gln	0,002230	one way ANOVA
	EV2G-YUMM1.7 proliferation	P value	Statistical test
	48h-Gln vs 48h+Gln	0,046660	one way ANOVA
	72h-Gln vs 72h+Gln	0,006500	one way ANOVA
	EV2G-migrated YUMM1.7	P value	Statistical test
	Glufo 10 +Gln vs Glufo 10 -Gln	0,043000	one way ANOVA
	Gluf 20 +Gln vs veh -Gln	0,007600	one way ANOVA
	Gluf 20 +Gln vs Gluf 10 -Gln	0,008700	one way ANOVA
	Gluf 20 +Gln vs Gluf 20 -Gln	0,022100	one way ANOVA
	EV2H	P value	Statistical test
	veh vs gluf 20	0,009990	two tailed *t* test
	EV2O	P value	Statistical test
	veh vs gluf 20	0,005999	two tailed *t* test
Figure EV3	EV3C	P value	Statistical test
	veh/IgG vs 20 /AntiCSF1R	0,043400	two way ANOVA
	veh/IgG vs veh/AntiCSF1R	0,033300	two way ANOVA
	20 /IgG vs 20 /AntiCSF1R	0,011100	two way ANOVA
	20 /IgG vs veh /AntiCSF1R	0,038760	two way ANOVA
	EV3E	P value	Statistical test
	veh/IgG vs 20 /IgG	0,033440	one way ANOVA
	20 /IgG vs veh /AntiCSF1R	0,000540	one way ANOVA
	20 /IgG vs 20 /AntiCSF1R	0,000223	one way ANOVA
	EV3F	P value	Statistical test
	veh/IgG vs 20 /IgG	0,044400	one way ANOVA
	veh/AntiCSFR1 vs 20/AntiCSF1R	0,022230	one way ANOVA
