# Supplementary material for: Glufosinate constrains synchronous and metachronous metastasis by promoting anti‐tumor macrophages
Source: EMBO Mol Med. 2020 Sep 4;12(10):e11210. doi: 10.15252/emmm.201911210 (PMC7539200; doi:10.15252/emmm.201911210)
Supplement: Supplementary file 2 — Expanded View Figures PDF [file EMMM-12-e11210-s002.pdf]

Expanded View Figures

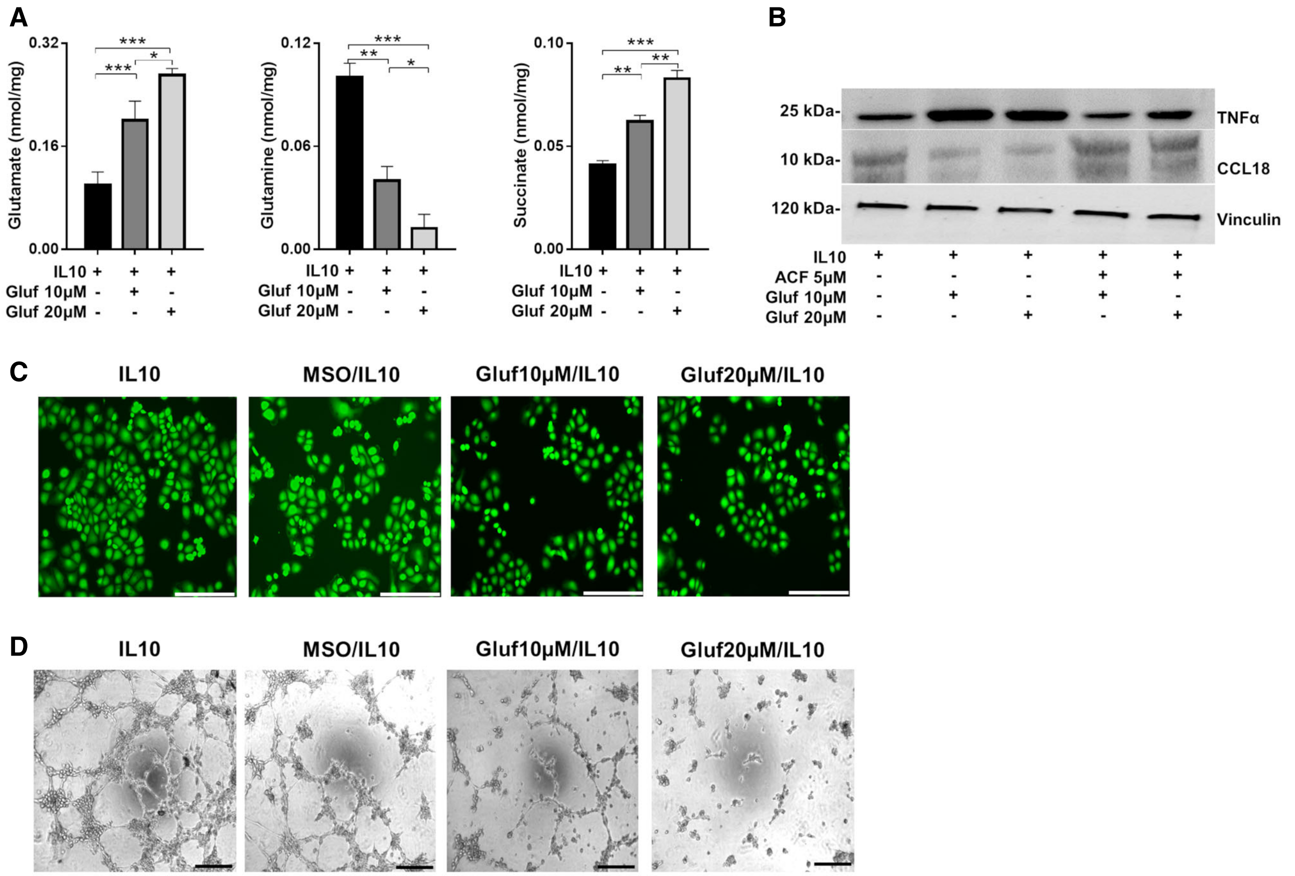

Figure EV1. Glufosinate triggers a metabolic and functional switch of macrophages toward an M2-like phenotype.

A LC-MS/MS quantification of intracellular glutamate, glutamine, and succinate in IL10, MSO-, and glufosinate (10 and 20 μM)-treated IL10 (n = 4).

B Western blotting analysis of TNFα and CCL18 in IL10 alone or glufosinate (10 and 20 μM)- and acriflavine/glufosinate (10 and 20 μM)-IL10 macrophages (n = 3).

C Representative images of A549 cell migration through a matrigel-coated micropore filter in presence of IL10, MSO/IL10, and glufosinate (10 and 20 μM)-IL10 pre-stimulated macrophages after 24 h of incubation (n = 6). Five images per field were analyzed. Scale bar: 50 μm.

D Representative pictures of capillary network formation of HUVEC cells cocultured with macrophages pretreated for 24 h with IL10 or MSO/IL10, and glufosinate (10 and 20 μM)/IL10 after 4 h of incubation with HUVEC cells (n = 6). Five images per field were analyzed. Scale bar: 100 μm.

Data information: Data are reported as means ± SEM. *$P < 0.05$, **$P < 0.01$, ***$P < 0.001$, ****$P < 0.0001$. Exact P values and statistical tests are reported for each experiment in Appendix Table S2.
Source data are available online for this figure.

© 2020 The Authors

Figure EV2. Glufosinate skews macrophages toward an M2-like phenotype but does not affect cancer cell proliferation.

A, B RT–PCR quantification of M1 or M2 markers in murine BMDMs. (A) Fold increase of *Tnfα*, *Cxcl10*, and *Nos2* mRNA in IL10, glufosinate (10 and 20 μM)-IL10 macrophages for 24 h ($n = 3$). (B) Fold reduction of *Arg1* and *Ccl22* mRNA in macrophages as above ($n = 3$).

C Evaluation in a transwell system of murine lung cancer cells proliferation (LLC) in GLN-free medium, alone (Mφ-) or in presence of murine IL10 polarized BMDMs, pretreated or not with glufosinate (10 and 20 μM) ($n = 6$).

D Evaluation of murine lung cancer cells (LLC) migrating through a matrigel layer, under the stimulus of murine IL10 and glufosinate (10 and 20 μM)-IL10 BMDMs ($n = 6$).

E–G Evaluation of murine lung cancer cells (LLC) (E), breast cancer cells (4T1) (F), and melanoma cancer cells (YUMM1.7) (G) proliferation and migration through a matrigel-coated micropore filter in presence of glufosinate or vehicle, in normal and glutamine deprived media ($n = 6$).

H Quantification of glutamine (GLN) levels in plasma of vehicle and glufosinate-treated mice ($n = 8$), by liquid chromatography–mass spectrometry (LC-MS).

I Western blotting analysis and densitometric analysis of GS in LLC tumor from vehicle and glufosinate-treated mice. Representative lanes of the same Western blot run and exposure (pool of 2 independent experiments; 10 mice per condition).

J Evaluation of LLC tumor weight in vehicle and glufosinate (10 and 20 mg/kg)-treated mice (pool of 2 independent experiments; 10 mice per condition in total).

K, L Quantification of F4/80$^+$ macrophage density in metastatic niche (K) and in tumors (L) of vehicle and glufosinate-treated LLC-bearing mice ($n = 6$).

M FACS evaluation of CD4$^+$ T cells in vehicle and glufosinate (10 and 20 mg/kg)-treated mice ($n = 4$).

N, O Tumor weight (N) and metastatic count after india ink injection (O) in vehicle and 20 mg/kg glufosinate ammonium-treated LLC-bearing C57Bl/6N mice (pool of 2 independent experiments; 10 mice per condition in total).

Data information: Data are reported as means ± SEM. *$P < 0.05$, **$P < 0.01$, ***$P < 0.001$, ****$P < 0.0001$. Exact P values and statistical tests are reported for each experiment in Appendix Table S2.
Source data are available online for this figure.

 © 2020 The Authors

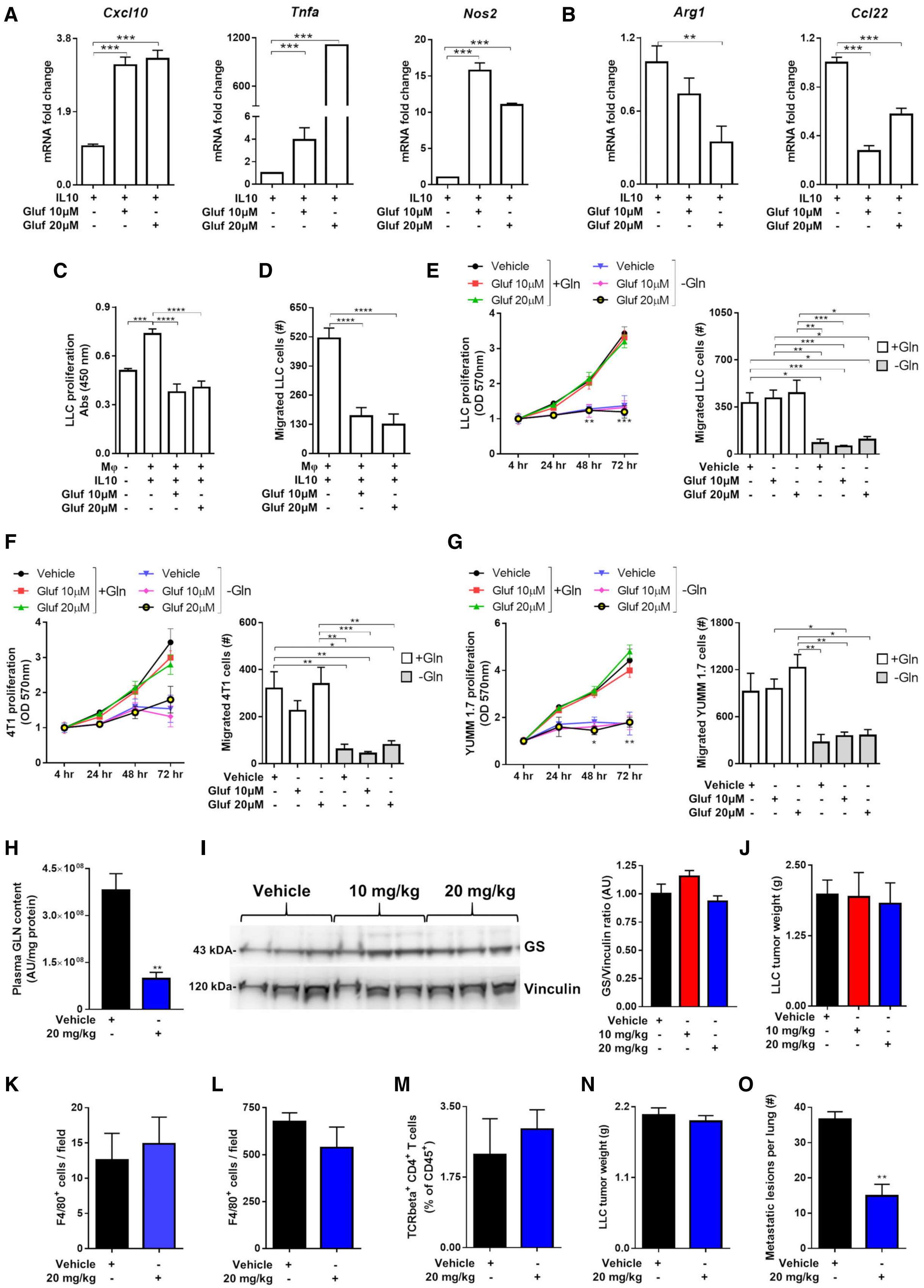

A
Cxcl10
Tnfa
Nos2
B
Arg1
Ccl22
mRNA fold change
IL10
Gluf 10μM
Gluf 20μM
C
D
E
LLC proliferation Abs (450 nm)
Migrated LLC cells (#)
Mφ
Vehicle
Gluf 10μM
Gluf 20μM
+Gln
-Gln
LLC proliferation (OD 570nm)
4 hr
24 hr
48 hr
72 hr
F
G
4T1 proliferation (OD 570nm)
Migrated 4T1 cells (#)
YUMM 1.7 proliferation (OD 570nm)
Migrated YUMM 1.7 cells (#)
H
I
J
Plasma GLN content (AU/mg protein)
20 mg/kg
10 mg/kg
43 kDA-
120 kDa-
GS
Vinculin
GS/Vinculin ratio (AU)
LLC tumor weight (g)
K
L
M
N
O
F4/80+ cells / field
TCRbeta+ CD4+ T cells (% of CD45+)
Metastatic lesions per lung (#)

Figure EV2.

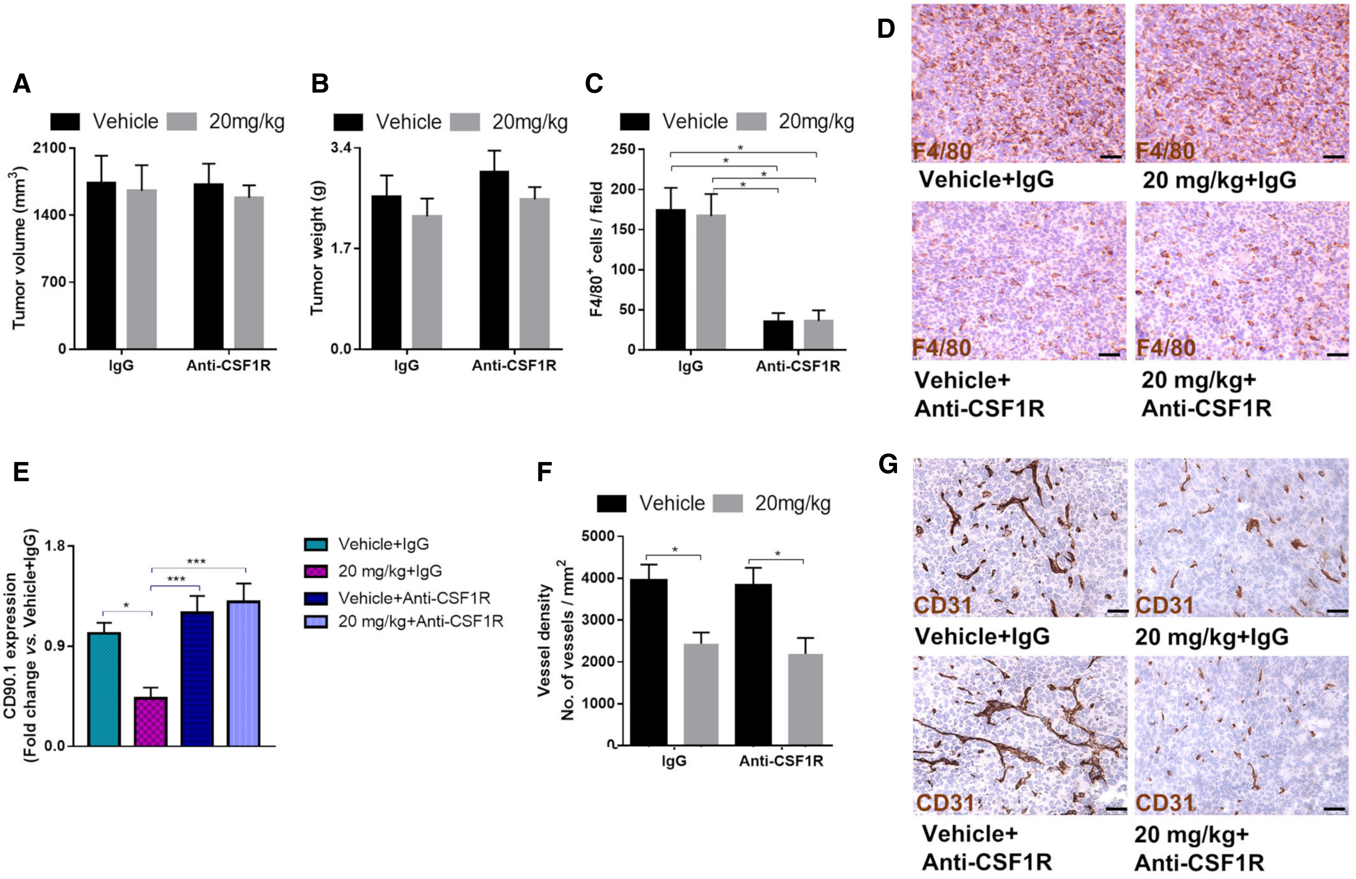

Figure EV3. Upon *in vivo* macrophage depletion, glufosinate loses its antimetastatic effect but still constrains tumor vessel formation.

A, B Evaluation of tumor volume (A) and weight (B) in LLC ($CD90.1^+$)-tumor-bearing mice preconditioned with IgG or anti-CSF1R antibody and treated with vehicle or glufosinate (20 mg/kg).

C, D Quantification (C) and representative images (D) of $F4/80^+$ macrophage infiltration in LLC ($CD90.1^+$)-tumors from mice preconditioned with IgG or anti-CSF1R antibody and treated with vehicle or glufosinate (20 mg/kg). Six images per tumor were analyzed. Scale bar: 50 μm.

E $CD90.1^+$ CTCs, extrapolated by measuring CD90.1 expression level by qPCR of cDNAs obtained from the blood of LLC ($CD90.1^+$) tumor-bearing mice preconditioned with IgG or anti-CSF1R antibodies and treated with vehicle or glufosinate (20 mg/kg).

F, G Quantification (F) and representative images (G) of vessel density in LLC ($CD90.1^+$) tumors from mice preconditioned with IgG or anti-CSF1R antibody and treated with vehicle or glufosinate (20 mg/kg). Six images per tumor were analyzed. Scale bar: 50 μm.

Data information: Data are reported as means ± SEM. *$P < 0.05$, **$P < 0.01$, ***$P < 0.001$, ****$P < 0.0001$. Exact *P* values and statistical tests are reported for each experiment in Appendix Table S2.

 © 2020 The Authors

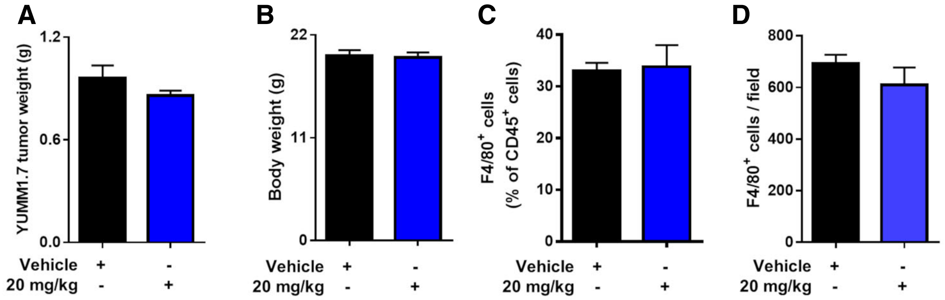

Figure EV4. Glufosinate treatment does not affect tumor weight, body weight, and TAM's infiltration in a murine melanoma model.

A, B Evaluation of tumor (A) and body weight (B) in YUMM 1.7-tumor-bearing mice treated with vehicle or glufosinate (20 mg/kg) (pool of 2 independent experiments; 10 mice per condition in total).

C FACS quantification of F4/80$^+$ cells in tumors of vehicle and glufosinate-treated mice (n = 6).

D Quantification of F4/80$^+$ macrophage density in tumors of vehicle and glufosinate-treated YUMM 1.7-tumor-bearing mice (n = 6).

Data information: Data are reported as means ± SEM. *P < 0.05, **P < 0.01, ***P < 0.001, ****P < 0.0001.

© 2020 The Authors
