# Supplementary figures and images for: Glufosinate constrains synchronous and metachronous metastasis by promoting anti‐tumor macrophages
Source: EMBO Mol Med. 2020 Sep 4;12(10):e11210. doi: 10.15252/emmm.201911210 (PMC7539200; doi:10.15252/emmm.201911210)

Source data Fig. EV1

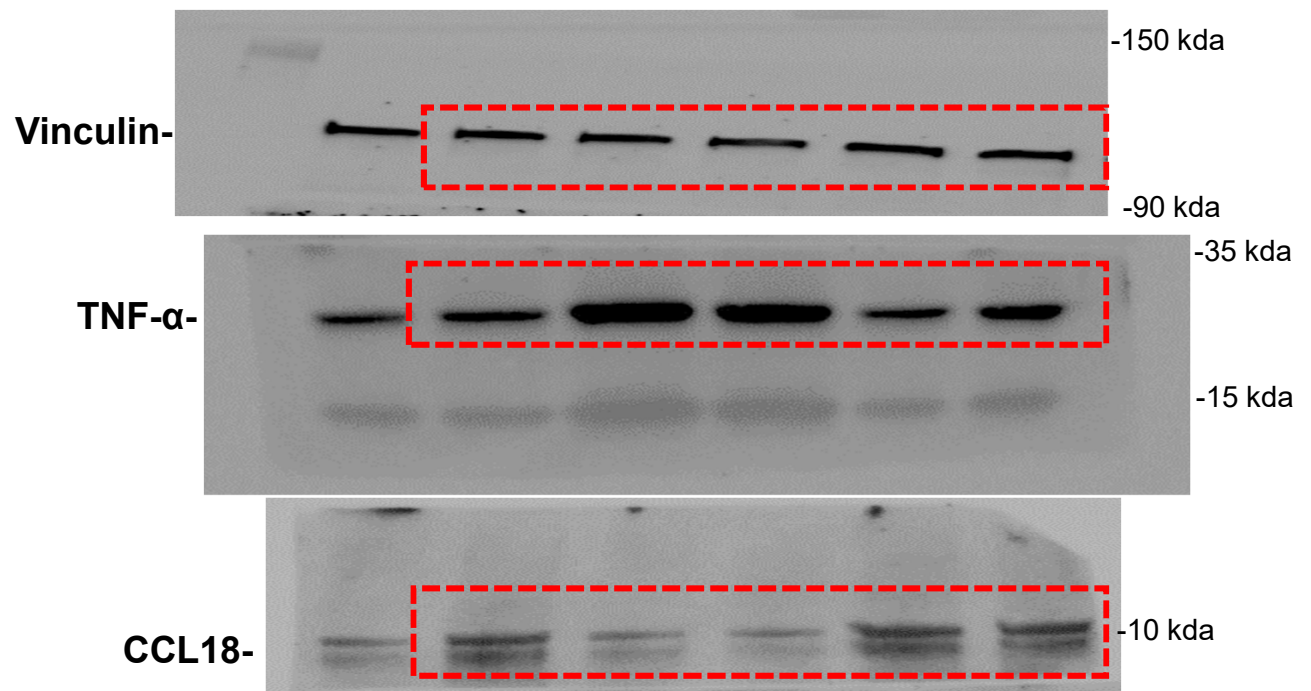

Supplement: Supplementary file 6 — Source Data for Expanded View [file EMMM-12-e11210-s014.zip › Source_Data_EV1.pdf]

Full unedited blot for Figure EV2

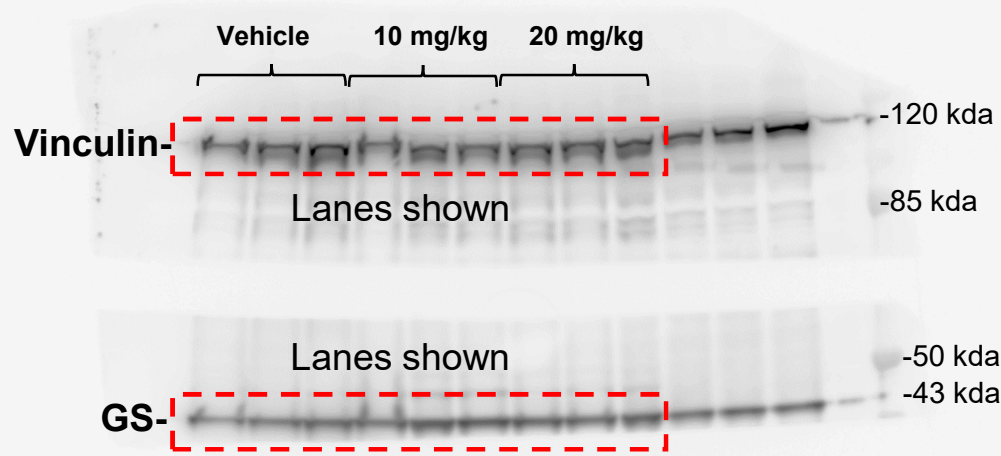

Supplement: Supplementary file 6 — Source Data for Expanded View [file EMMM-12-e11210-s014.zip › Source_Data_EV2.pdf]

Source data Fig. 1

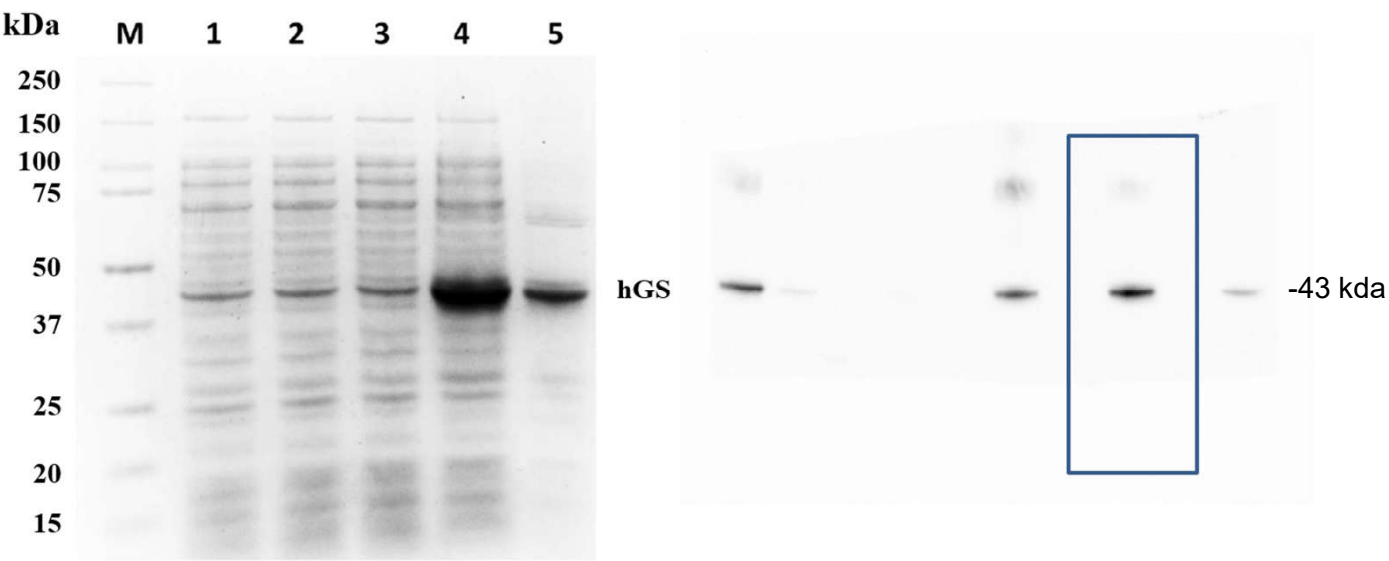

Supplement: Supplementary file 8 — Source Data for Figure 1 [file EMMM-12-e11210-s006.pdf]

Full unedited blot for Figure 2U

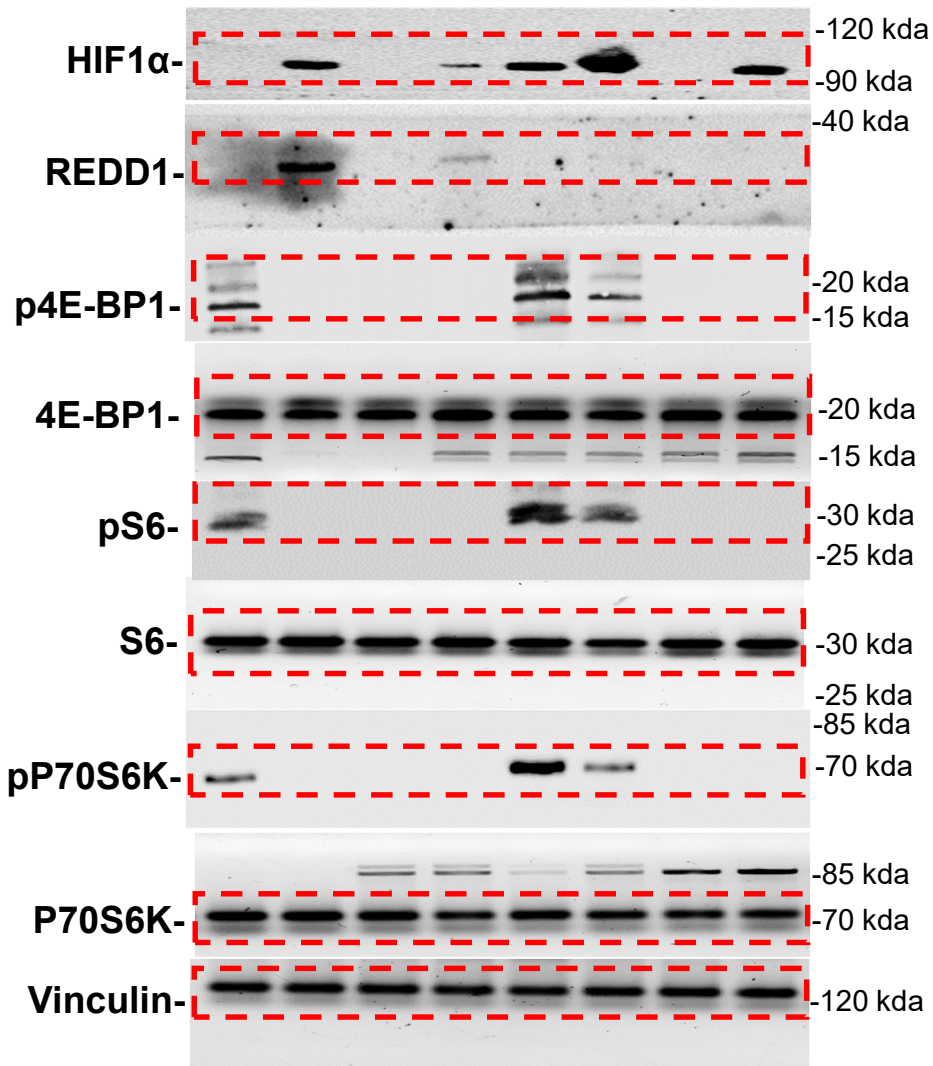

Supplement: Supplementary file 9 — Source Data for Figure 2 [file EMMM-12-e11210-s007.pdf]

Source Data – Figure 3F

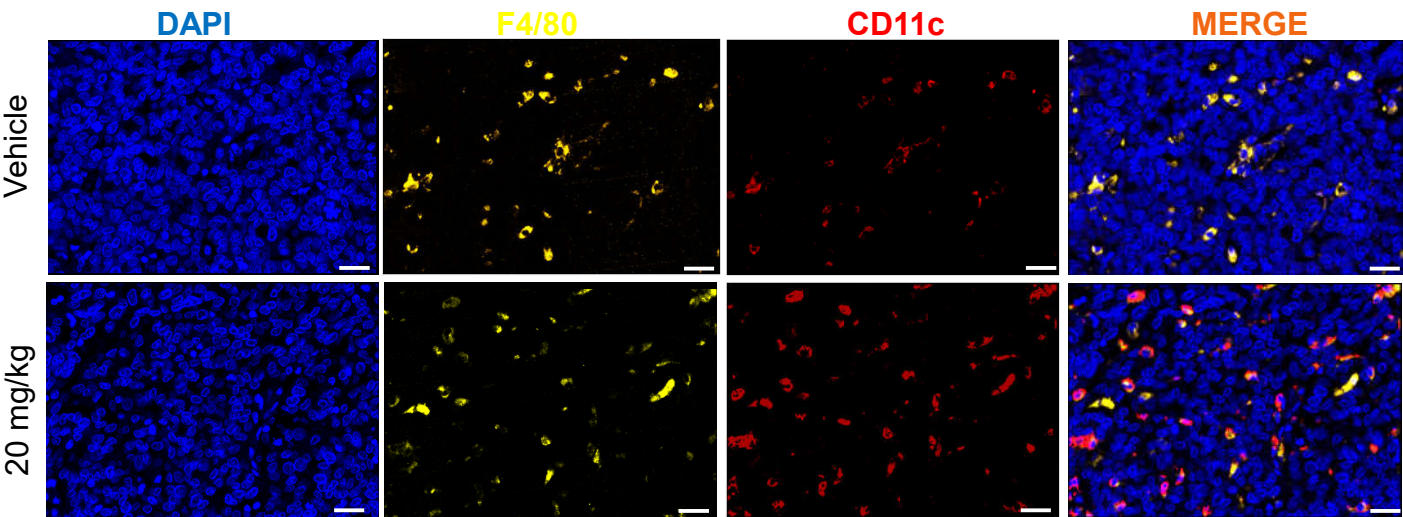

Source Data – Figure 3G

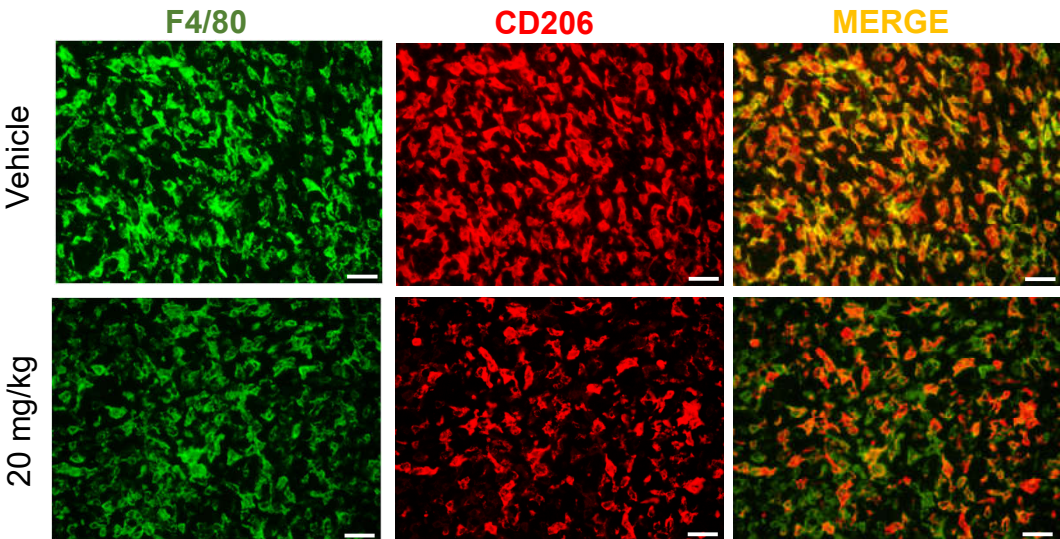

Source Data – Figure 3H

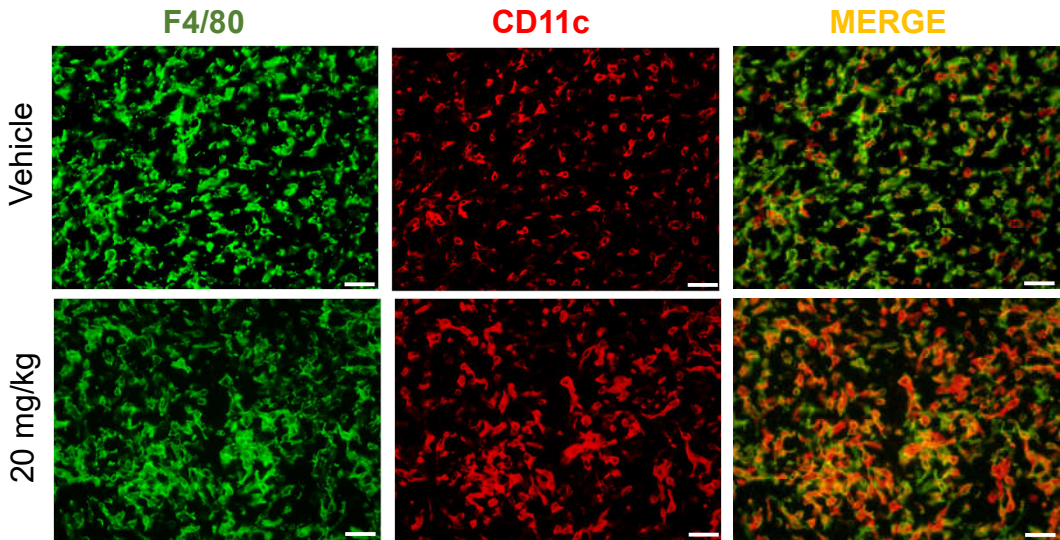

Supplement: Supplementary file 10 — Source Data for Figure 3 [file EMMM-12-e11210-s008.pdf]

Source Data – Figure 4C

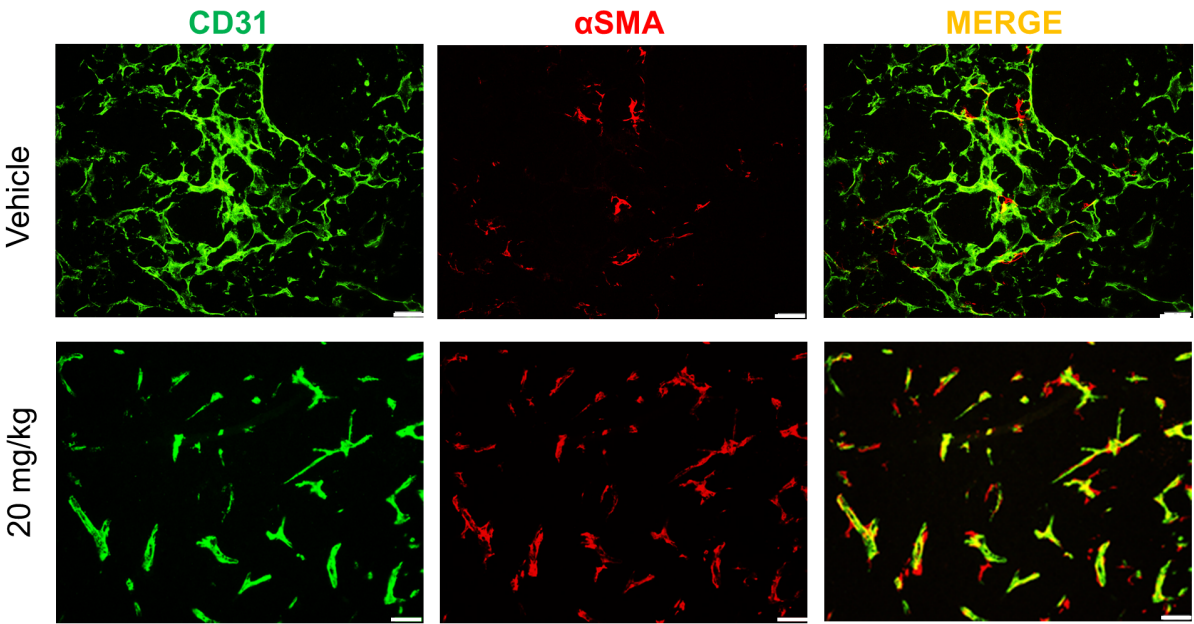

Source Data – Figure 4D

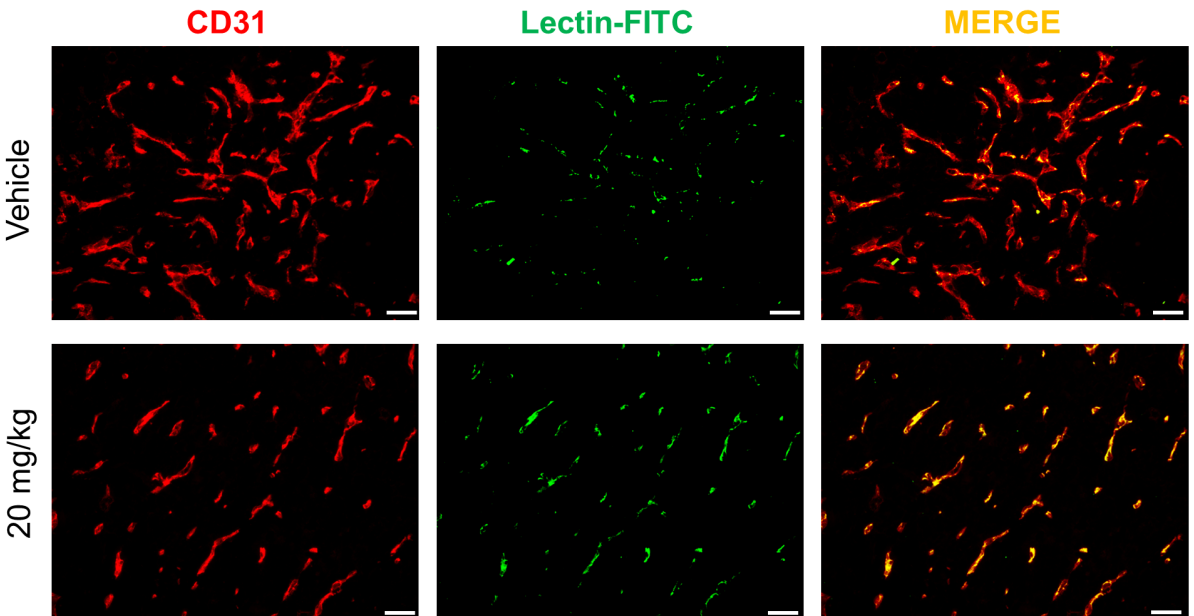

Supplement: Supplementary file 11 — Source Data for Figure 4 [file EMMM-12-e11210-s009.pdf]

Source Data – Figure 5C

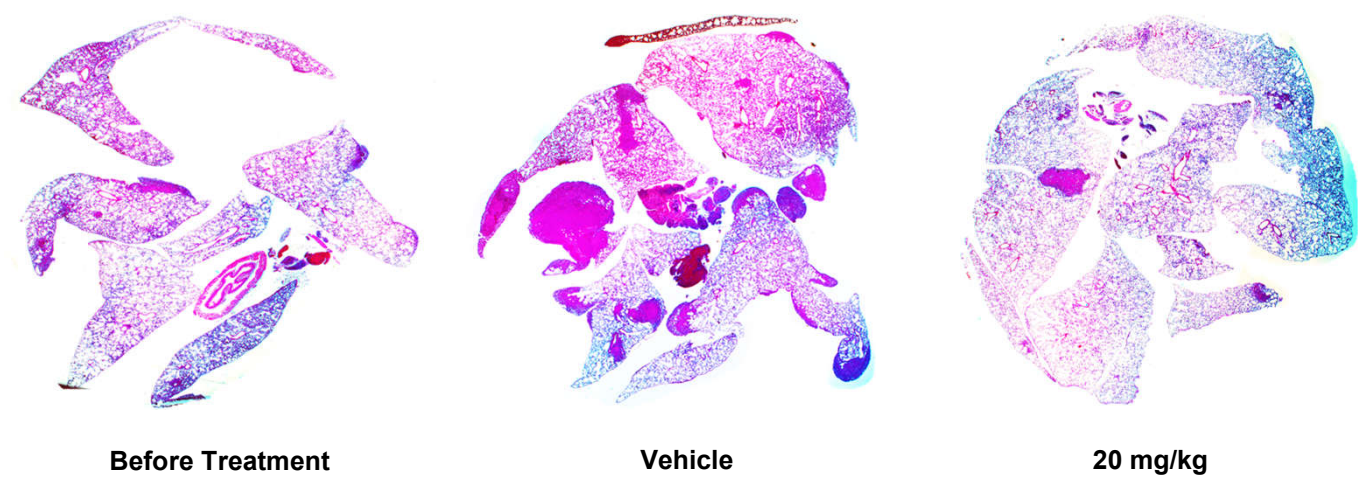

Source Data – Figure 5G

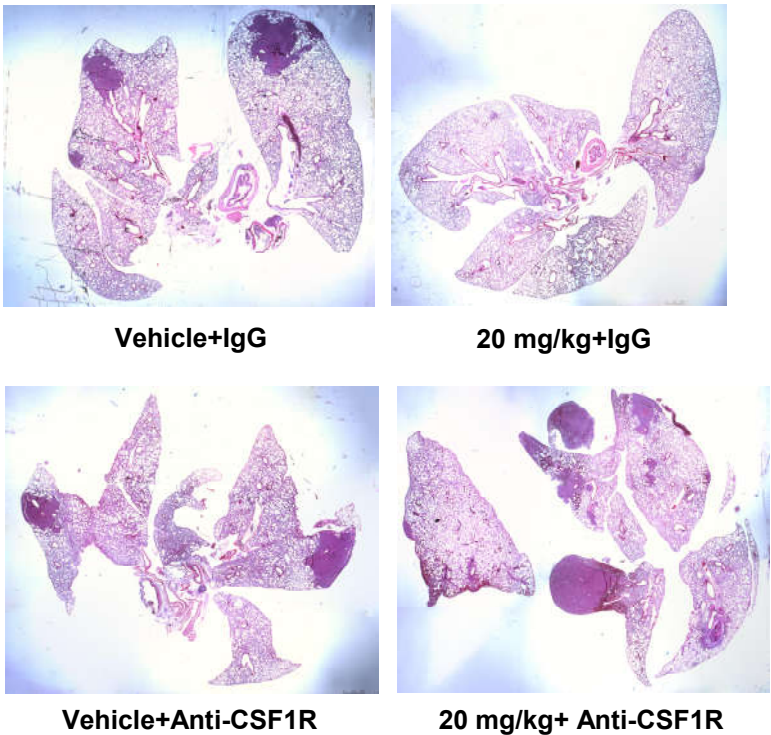

Supplement: Supplementary file 12 — Source Data for Figure 5 [file EMMM-12-e11210-s010.pdf]

Source Data – Figure 6D

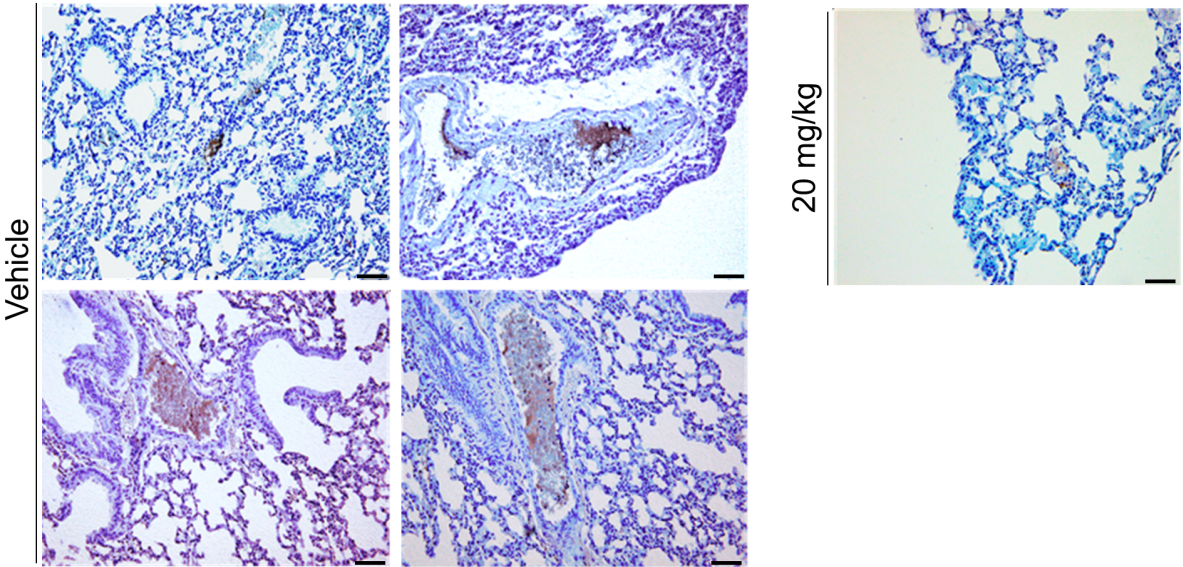

Source Data – Figure 6G

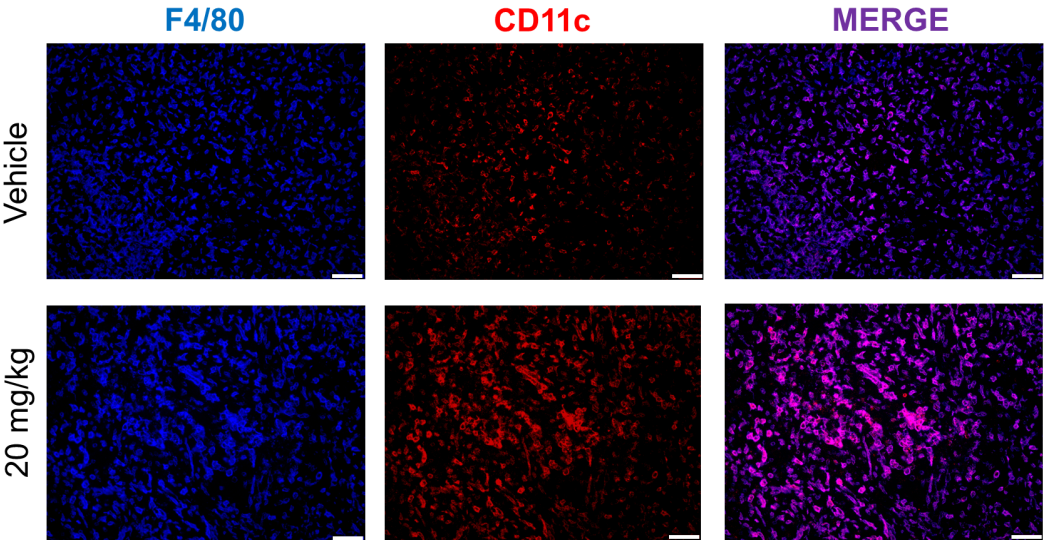

Source Data – Figure 6J

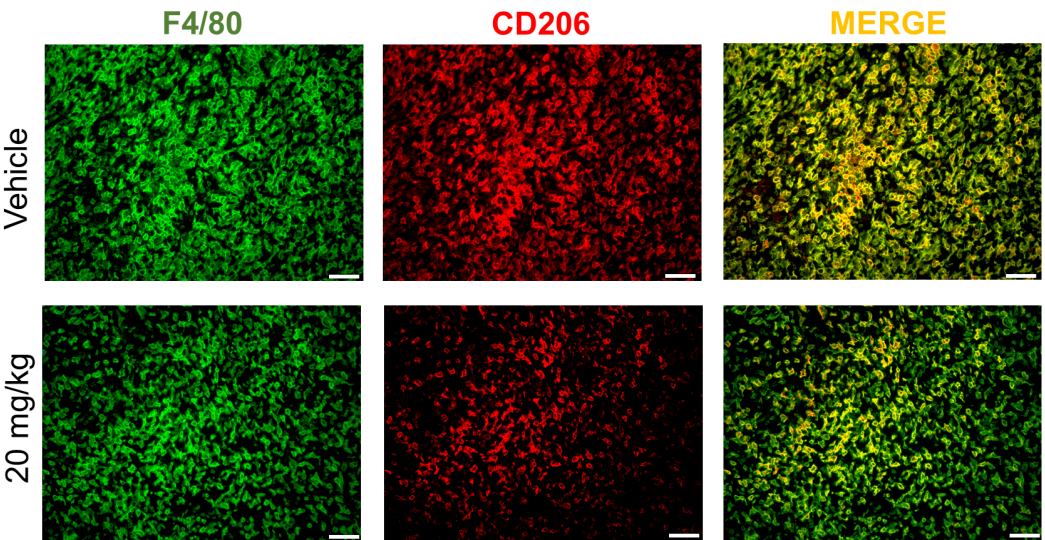

Supplement: Supplementary file 13 — Source Data for Figure 6 [file EMMM-12-e11210-s011.pdf]

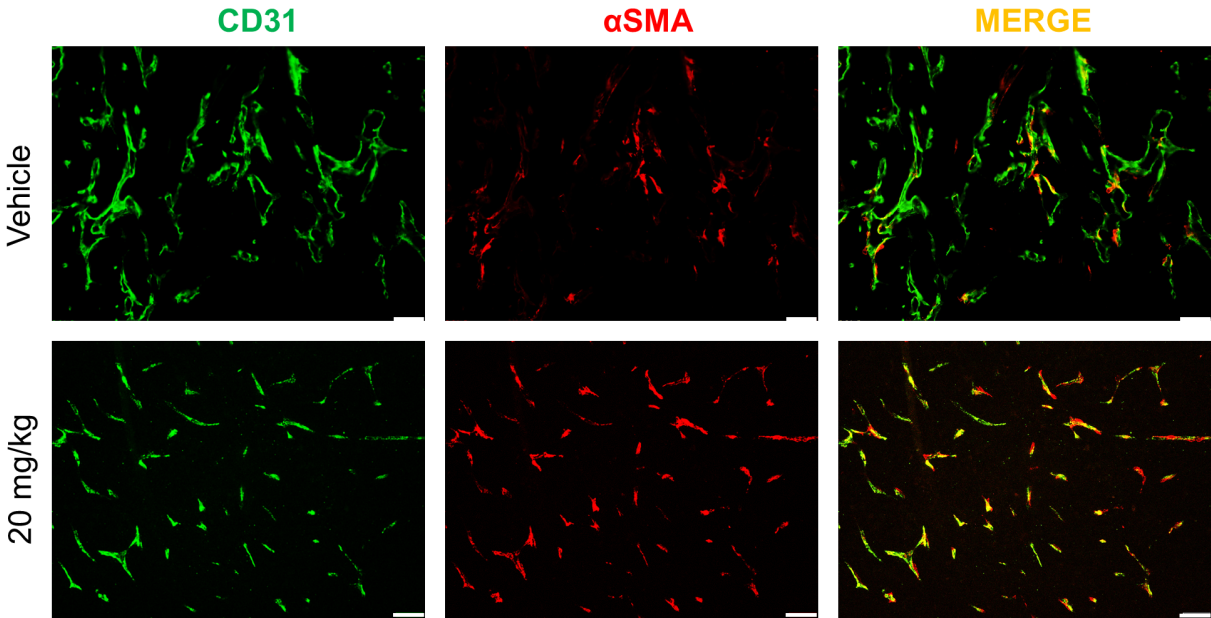

Supplement: Supplementary file 14 — Source Data for Figure 7 [file EMMM-12-e11210-s012.pdf]

Source Data – Figure 8G

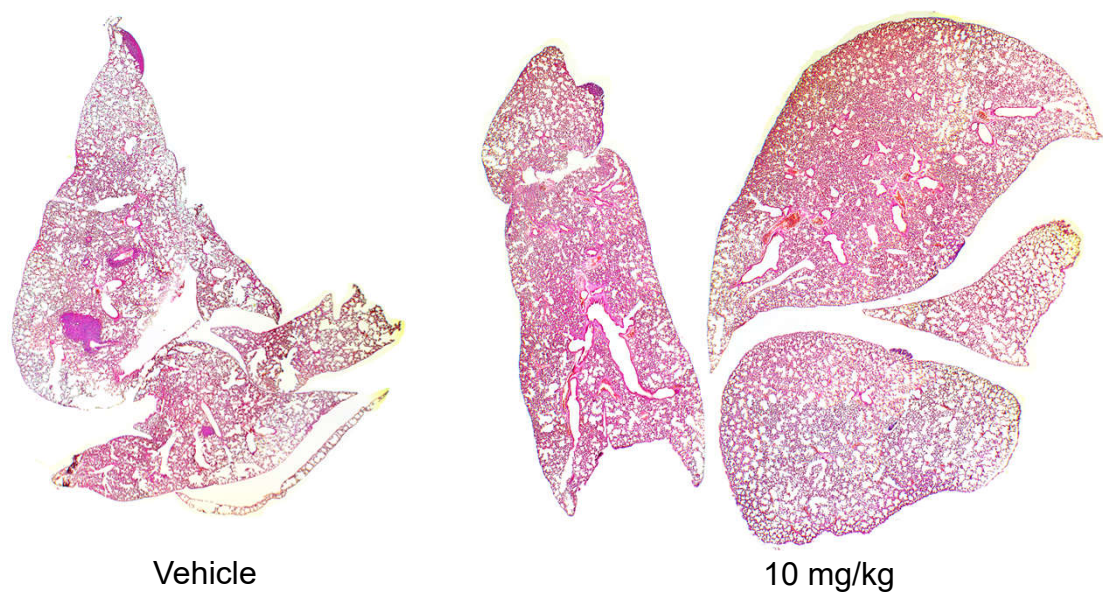

Source Data – Figure 8I

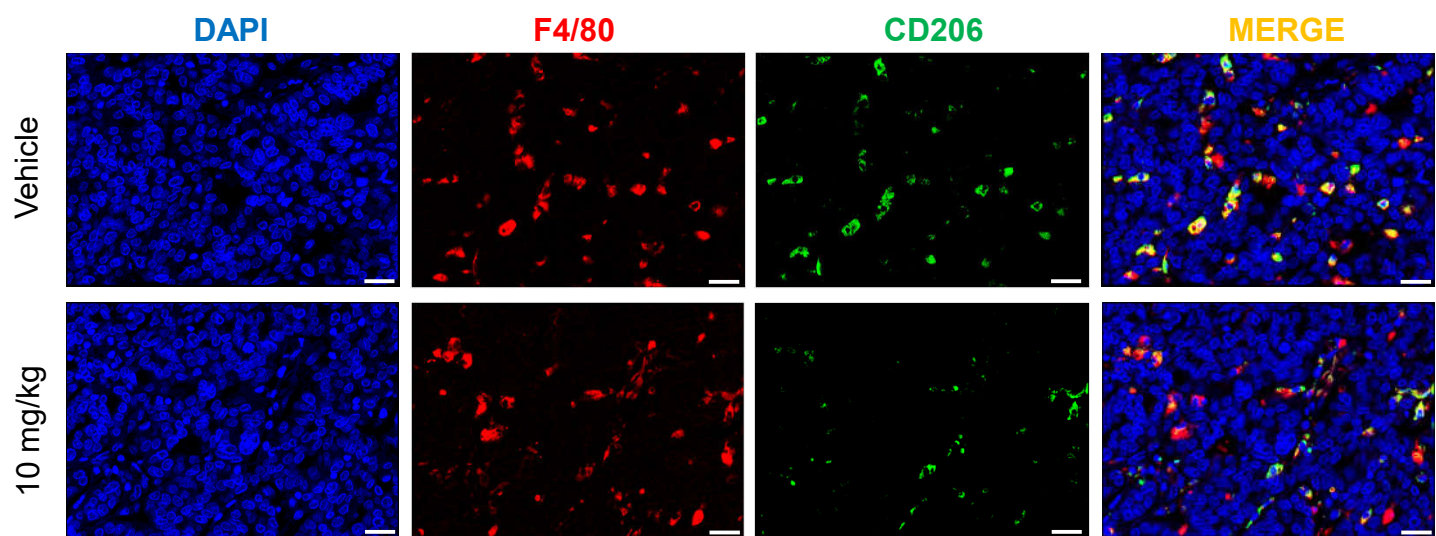

Supplement: Supplementary file 15 — Source Data for Figure 8 [file EMMM-12-e11210-s013.pdf]
